# Supplementary material for: Antiviral Properties of Pennisetum purpureum Extract against Coronaviruses and Enteroviruses
Source: Pathogens. 2022 Nov 17;11(11):1371. doi: 10.3390/pathogens11111371 (PMC9696772; doi:10.3390/pathogens11111371)
Supplement: Supplementary file 1 [file pathogens-11-01371-s001.zip › pathogens-2004435-supplementary.pdf]

Supplementary Materials

Table S1 shows the Antiviral effect of *Pennisetum purpureum* extract (Heyiya®) against enterovirus 71 (EV71), feline coronavirus (FCoV), and porcine epidemic diarrhea virus. Virus titers ($TCID_{50}$/100μL) of the viruses pretreated with 10-fold serial dilutions of the extract for 1 hour (1h) and 6 hours (6h) were determined by $TCID_{50}$ assay. The viruses without pretreatment of the extract were used as the positive control (EV71, FCoV, PEDV). Relative $TCID_{50}$/100μL to control was calculated as follow: $\log_{10}$ ($TCID_{50}$/100μL of the virus without pretreatment of the extract)-$\log_{10}$ ($TCID_{50}$/100μL of the virus pretreated with the extract),

Table S1. Virus titers ($TCID_{50}$/100μL) of the viruses without pretreatment (EV71, FCoV, PEDV) or the viruses pretreated with 10-fold serial dilutions of the *Pennisetum purpureum* extract (Heyiya®) for 1 hour and 6 hours

Groups	$Log_{10}TCID_{50}$/100μL					
	R1	R2	R3	Average	STEDV	Relative to control
EV71, 1h	10	10	10	10	0	
EV71, 6h	8	8.5	10	9.54	1.04	
EV71+1/100 Heyiya®, 1h	10	9	10	9.85	0.58	-0.15
EV71+1/100 Heyiya®, 6h	7	6.4	5.8	6.64	0.6	-3.36
EV71+1/1000 Heyiya®, 1h	10	7.25	10	9.82	1.59	-0.18
EV71+1/1000 Heyiya®, 6h	7.25	6.67	10	9.52	1.78	-0.48
EV71+1/10000 Heyiya®, 1h	9.5	7.25	9	9.14	1.18	-0.86
EV71+1/10000 Heyiya®, 6h	10	7.5	8	9.53	1.32	-0.47
FCoV, 1h	4	3.57	3.8	3.79	0.22	
FCoV, 6h	3.67	3.67	3.67	3.67	0	
FCoV+1/10 Heyiya®, 1h	5	4.25	3.8	4.62	0.61	+0.83
FCoV+1/10 Heyiya®, 6h	2.5	2.57	2.8	2.57	0.19	-1.22
FCoV+1/100 Heyiya®, 1h	3.57	4.4	4.5	4.3	0.51	+0.51
FCoV+1/100 Heyiya®, 6h	3	3	2.67	2.92	0.51	-0.75
FCoV+1/1000 Heyiya®, 1h	5.25	4.5	4	4.86	0.63	+1.07
FCoV+1/1000 Heyiya®, 6h	4.5	3.8	3.5	4.17	0.51	+0.38
PEDV, 1h	3.5	3.8		3.68	0.21	
PEDV, 6h	2.33	2.5		2.42	0.12	
PEDV+1/10 Heyiya®, 1h	2	1.8		1.91	0.14	-1.77
PEDV+1/10 Heyiya®, 6h	0	0		0	0	-3.68
PEDV+1/100 Heyiya®, 1h	3.25	4.33		4.06	0.76	+0.38
PEDV+1/100 Heyiya®, 6h	2.67	3		2.87	0.23	-0.81
PEDV+1/1000 Heyiya®, 1h	3.42	3.5		3.46	0.06	-0.22
PEDV+1/1000 Heyiya®, 6h	2.5	2.57		2.54	0.05	-1.14

Table S2. The body length (cm) of chicken embryo inoculated with infectious bronchitis virus (IBV) without the pretreatment of *Pennisetum purpureum* extract (Heyiya®), or with IBV pretreated with 10-fold or 100-fold diluted extract for 1h or 6h at room temperature, or without IBV (NC)

	Embryo body length (cm)						
	R1	R2	R3	R4	R5	Average	STEDV
IBV	5.5	4	4.5			4.67	0.76
IBV+1/10 Heyiya®, 6h	6.6	6.6	6.5	5.1	6.1	6.18	0.64
IBV+1/10 Heyiya®, 1h	4.6	4.3	6.1			5	0.96
IBV+1/100 Heyiya®, 6h	5	5.5	5.7	5	5	5.24	0.34
IBV+1/100 Heyiya®, 1h	5.2	5.2	5.7	4.3	5.4	5.16	0.52
NC (without IBV)	7	7	7			7	0
